# Comparison of Digital versus Analog ^68^Ga-PSMA-11 PET/CT Performance in Hormone-Sensitive Prostate Cancer Patients with Early Biochemical Recurrence or Persistence after Radical Treatment

**DOI:** 10.3390/diagnostics13233535

**Published:** 2023-11-26

**Authors:** Guido Rovera, Serena Grimaldi, Sara Dall’Armellina, Michela Zotta, Monica Finessi, Roberto Passera, Désirée Deandreis

**Affiliations:** 1 Nuclear Medicine Division, Department of Medical Sciences, University of Turin, 10126 Turin, Italy; guido.rovera@unito.it; 2 Nuclear Medicine Division, Department of Medical Sciences, AOU Città della Salute e della Scienza di Torino, University of Turin, 10126 Turin, Italy; 3 Nuclear Medicine Division, Gustave Roussy, 94805 Villejuif, France

**Keywords:** PET/CT, digital PET/CT, ^68^Ga-PSMA-11, prostate cancer, biochemical recurrence

## Abstract

The aim of this study was to investigate whether the favorable characteristics of novel digital PET/CT (dPET) scanners compared to analog systems (aPET) could translate into an improved disease localization in prostate cancer (PCa) patients with early biochemical recurrence/persistence (BCR/BCP). A retrospective analysis was conducted on 440 consecutive analog (*n* = 311) or digital (*n* = 129) ^68^Ga-PSMA-11 PET/CT scans performed in hormone-sensitive ADT-free PCa patients with early-BCR/BCP (PSA at PET ≤ 2.0 ng/mL), previously treated with radical intent (radical-prostatectomy/radiotherapy). dPET showed a higher positivity rate compared to aPET (48.8% [63/129] vs. 37.3% [116/311], *p* = 0.03), despite the slightly lower median PSA value of the dPET cohort (0.33 [IQR: 0.26–0.61] vs. 0.55 [IQR: 0.40–0.85] ng/mL, *p* < 0.01). dPET detection rate was higher in both PSA ranges 0.2–0.5 ng/mL (39.0% [32/82] vs. 25.2% [34/135], *p* = 0.03) and 0.5–1.0 ng/mL (63.2% [24/38] vs. 40.8% [53/130], *p* = 0.02), but not for PSA ≥ 1.0 ng/mL. dPET detected a higher per patient median number of pathologic findings (PSMA-RADS ≥ 3) and multi-metastatic cases (>3 lesions) among N1/M1-positive scans (21.7% [10/46] vs. 8.6% [9/105], *p* = 0.03). Moreover, the proportion of uncertain findings among pathological lesions was significantly lower for dPET than aPET (24.4% [39/160] vs. 38.5% [60/156], *p* = 0.008). Overall, ^68^Ga-PSMA-11 dPET showed a better performance compared to aPET, resulting in a higher scan-positivity rate, a higher number of detected pathological lesions, and a lower rate of uncertain findings.

## 1. Introduction

The clinical management of prostate cancer (PCa) patients with biochemical recurrence (BCR) or persistence (BCP) requires accurate lesion localization and precise disease restaging. The performance of conventional imaging (i.e., computed tomography [CT] and bone scintigraphy) in localizing disease recurrence is suboptimal compared to molecular hybrid imaging [1,2,3,4]. However, radiotracers such as ^18^F-fluciclovine and ^18^F-choline cannot achieve a high diagnostic accuracy in patients with early PCa recurrence and low prostate-specific antigen (PSA) values (<2.0 ng/mL) [5,6]. Recently, a new class of radiopharmaceuticals which targets the prostate-specific membrane antigen (PSMA) has become available. PSMA is a transmembrane glycoprotein constitutively expressed within the apical epithelium of prostatic secretory ducts and overexpressed in prostate cancer cells, in which it migrates to the luminal surface as malignant transformation occurs.

Prostate-specific membrane antigen/positron emission tomography (PSMA-PET) has shown a good performance in restaging BCR and BCP patients, allowing the early localization of loco-regional and/or distant metastases [7,8,9,10,11]. Currently, the European Association of Urology (EAU) guidelines recommend performing a PSMA-PET scan in patients with PSA failure after radical treatment, provided that the imaging data can potentially impact the patient’s clinical management [12]. Indeed, the early and precise identification of metastatic involvement at molecular imaging with PSMA-PET could significantly alter the clinical decision-making and therapeutic approach [13,14,15], leading to more personalized treatments. Literature evidence has shown PSMA-PET to be able to guide stereotactic ablative radiotherapy/stereotactic body radiation therapy (SABR/SBRT) in patients with a lower tumor burden and oligometastatic disease [16,17,18,19]. In particular, in the ORIOLE phase II trial, patients treated with SABR who received consolidation of all the disease localizations detected by PSMA-PET (baseline data blinded by protocol) showed a higher median progression-free survival (PFS unreached at 24 months follow-up vs. 11.8 months; HR 0.26; 95% CI: 0.09–0.76; *p* = 0.006) and a higher distant metastasis-free survival (29.0 vs. 6.0 months; HR 0.19; 95% CI: 0.07–0.54; *p* < 0.001) [19]. A pooled analysis of data from the ORIOLE and STOMP [20] trials also reported a sustained clinical benefit for metastasis-directed treatment (MDT) over observation in oligometastatic hormone-sensitive PCa patients [21]. Other studies have also reported promising results for PSMA-PET guided SBRT in terms of biochemical response [18,22], metastases-free survival (MFS) and castration-resistant-PCa-free survival (CRPC-FS) [23], distant progression-free survival (DPFS) [24], as well as overall survival [18]. This evidence supports the potential benefit of accurately restaging PCa patients with molecular imaging to allow for a metastasis-directed treatment (MDT) of oligometastatic spread. However, despite the availability of novel PSMA radioligands, the identification of locoregional and distant metastases at the earlier stages of biochemical recurrence still represents a significant challenge that drives nuclear medicine to improve the capabilities of molecular imaging towards an optimal diagnostic performance in the early stages of disease relapse [7].

In parallel with the recent discoveries in the field of radiopharmaceuticals, significant progress has also been made regarding PET/CT technology, leading to the introduction of a new generation of fully digital PET/CT (dPET) scanners based on solid state detectors, as opposed to previous generation analog PET (aPET) scanners based on photomultiplier tubes (PMT) coupled to multiple scintillation crystals. Digital PET/CT scanners (i.e., Philips Healthcare Vereos [25], GE Healthcare Discovery MI [26], and Siemens Healthineers Biograph Vision [27]) allow for significant technical improvements including 1:1 coupling of crystals and detectors, improved spatial resolution, shorter dead-time, faster time of flight (TOF), better TOF reconstruction methods, and an enhanced signal-to-noise ratio.

The technological improvements introduced by fully digital PET/CT scanners also include more advanced reconstruction algorithms such as Bayesian penalized-likelihood iterative image reconstruction algorithms that offer the advantage of reaching full convergence without introducing excessive noise as the number of iterations increases [28].

The superior technical capabilities of digital PET/CT systems compared to analog scanners hold potential for an earlier and more accurate identification of smaller lesions and a more precise quantification of their tracer uptake, thus potentially leading to a higher detection rate and a higher likelihood of a change in the patient’s management. In high-risk prostate cancer patients with early biochemical recurrence or persistence after radical treatment, novel digital PET/CT systems could potentially better leverage the sensitivity of PSMA radioligands and help to identify oligometastatic patients eligible for SABR/SBRT treatments at an earlier stage of disease recurrence, when the tumor burden is lower and treatments are more effective.

Based on the previous considerations, the aim of this study was to investigate whether the favorable characteristics of a digital PET/CT (dPET) scanner compared to a previous generation analog system (aPET) could translate into an improved disease localization in hormone-sensitive prostate cancer patients with early biochemical recurrence/persistence (BCR/BCP) after radical treatment.

## 2. Materials and Methods

### 2.1. Study Design

A retrospective analysis was conducted on four hundred and forty (*n* = 440) consecutive ^68^Ga-PSMA-11 PET/CT scans performed at our institution (Department of Nuclear Medicine, University Hospital of Turin) with either an analog (11/2016–11/2020, *n* = 311) or digital (04/2021–10/2022, *n* = 129) scanner in hormone-sensitive ADT-free PCa patients with early biochemical recurrence or persistence (BCR/BCP, PSA at PET scan ≤ 2.0 ng/mL), previously treated with radical intent (radical prostatectomy/radiotherapy).

Inclusion criteria were: (1) histologically proven PCa; (2) previous treatment with radical intent, either radical prostatectomy (RP) or radiotherapy (RT); (3) proven biochemical recurrence (BCR) or biochemical persistence (BCP), as defined by the EAU guidelines [12], with PSA < 2.0 ng/mL; (4) hormone-sensitive prostate cancer (HSPC), in the absence of treatment with androgen deprivation therapy (ADT) in the 6 months prior to the PET scan. Exclusion criteria were: (1) patients not eligible for salvage therapy; (2) inability to undergo a PET/CT scan; (3) castration resistant PCa (CRPC); (4) concurrent administration of androgen-receptor targeted therapy or chemotherapy.

### 2.2. Procedures and Image Interpretation

The radiopharmaceutical (^68^Ga-PSMA-11) was synthesized in the radiochemistry laboratory of the Division of Nuclear Medicine of the AOU Città della Salute e della Scienza, University of Turin as previously documented [15], in accordance with procedure guidelines [29,30]. A ^68^Ga-PSMA-11 dose of 1.8–2.2 MBq/kg was injected intravenously to all patients followed by hydration with 0.5 L of saline solution during uptake. Informed consent was obtained from all subjects before administration. The diagnostic imaging did not require a specific patient preparation, and the subjects did not undergo the administration of furosemide or oral contrast media.

^68^Ga-PSMA-11 PET/CT imaging was performed according to standard recommendations, as previously described [15]. The images were acquired using either an analog (Gemini Dual, Philips HealthCare, Eindhoven, The Netherlands) or a digital (Vereos, Philips HealthCare, Eindhoven, The Netherlands) tomograph. PET emission data were co-registered with a low-dose CT scan for attenuation correction. In the analog PET/CT system, a low-dose CT scan with 120 kVp and 50 mAs/slice was acquired for attenuation correction. The reconstruction of PET images was conducted with the 3D row-action maximum likelihood algorithm (RAMLA) (4 iterations, 8 subsets). The field of view (FOV) size was 576 mm, the matrix size was 144 × 144, and the voxel size 4 mm^3^. In the digital PET/CT system, a low-dose CT scan with 120 kVp and automatic tube current modulation was acquired for attenuation correction. The reconstruction of PET images was conducted with the ordered subset expectation maximization (OSEM) algorithm (3 iterations, 15 subsets). The FOV size was 576 mm, the matrix size 144 × 144 and the voxel size 4 mm^3^.

The image evaluation was independently carried out by two experienced nuclear medicine physicians who reviewed all PET/CT images. Differences that emerged during image interpretation were settled through consensus. A per region analysis of PSMA-PET imaging was carried out as recommended by the European guidelines for PSMA-PET interpretation [30,31]. Pathological findings were defined as areas of increased focal radiopharmaceutical uptake compared to the background, not localized in sites of known physiologic uptake.

### 2.3. Statistical Analysis

For all individuals within the study, the gathered dataset was comprised of details regarding disease staging, histopathologic grading, prior treatments, PSA kinetics, and outcomes from PSMA-PET imaging. The uro-oncological tumor board (genitourinary oncology group, AOU Città della Salute e della Scienza, University Hospital of Turin, Italy) identified three distinct clinical scenarios pertaining to PSA failure as follows: (a) initial biochemical recurrence: PSA elevation of 0.2 ng/mL in case of previous RP or PSA levels ≥ 2 ng/mL above the nadir in case of primary RT; (b) PSA recurrence following salvage radiotherapy of the prostate bed: PSA elevation of ≥0.2 ng/mL over the nadir PSA level subsequent to prostate-bed salvage radiotherapy (SRT); (c) biochemical persistence after radical prostatectomy: PSA level ≥ 0.1 ng/mL 6 weeks after RP.

Anonymized data regarding patients’ clinical features, PSMA-PET results, and all the lesions identified at PET/CT imaging were stored and queried using a relational database [32]. Demographic characteristics of the population were described using absolute and relative frequencies for categorical variables, while for continuous variables, the median (Inter Quartile Range [IQR]) was employed. The determination of PSA doubling time followed the methodology established by Khan et al. [33], as previously outlined [15].

The cohorts were tested for homogeneity of baseline clinical parameters (e.g., T-stage ≥ 3a, ISUPgrade ≥ 3, radical treatment, time-to-recurrence, PSA doubling-time/velocity, adjuvant/salvage treatments). Inferential statistics were performed using the Mann–Whitney test for continuous covariates, and Fisher’s exact test for categorical ones, respectively. All reported *p*-values were two-sided, at the conventional 5% significance level. Data were analyzed as of August 2023 using IBM SPSS Statistics for Windows, version 26.0 (IBM Corp., Armonk, NY, USA).

## 3. Results

This retrospective analysis included four hundred and forty (*n* = 440) consecutive HSPC patients investigated with PSMA-PET at a single referral center (Nuclear Medicine, University Hospital of Turin—November 2016 to October 2022). Demographics and clinical characteristics of the study population are presented in Table 1.

The cohort median age was 75 (IQR: 70–80) years. The median PSA value at the time of the PSMA-PET scan was 0.51 (IQR: 0.33–0.80) ng/mL, while the median values for the aPET and dPET subgroups were 0.55 (IQR: 0.40–0.85) ng/mL and 0.33 (IQR: 0.26–0.61) ng/mL (*p* < 0.001), respectively. Regarding the PSA kinetic, the median PSA doubling time (PSAdt) of the whole cohort was 8.2 (IQR: 4.1–14.3) months. Overall, among patients with PSA failure, the clinical indications for PSMA-PET imaging were distributed as follows: 47.3% (208) first-time BCR, 38.2% (168) BCR after salvage-radiotherapy (SRT), and 14.5% (64) BCP cases.

The overall positivity rate for PSMA-PET imaging was 40.7% (179/440). According to the molecular imaging TNM (miTNM) definition [30,31], the distribution of the suspected PCa localizations in a per lesion analysis was as follows: prostate bed (miTr) in 7.7% of cases (34/440); pelvic nodes (miN1) in 21.8% (96/440); extra-pelvic nodes (miM1a) in 6.8% (30/440); bone metastasis (miM1b) in 10.7% (47/440); and visceral non-nodal metastasis (miM1c) in 2.3% (10/440). Considering the whole cohort in a per patient analysis, 22.7% (100/440) of cases presented with a locoregional recurrence (either prostate bed or pelvic lymph nodes), while 18.0% (79/440) showed a pathological involvement of at least one extra-pelvic region (either miM1a, miM1b or miM1c). Among metastatic patients (N/M+), the majority showed an oligometastatic spread (≤3 PSMA-positive localizations—87.4% [132/151]). Considering patients with a positive PET scan, the proportion of cases with distant metastatic involvement significantly varied among clinical settings, with fewer occurrences in first-time BCR (30.8%, 20/65) and higher frequencies in case of BCR after SRT (52.5%, 42/80) and BCP (50.0%, 17/34) (*p* = 0.02). The proportion of multi-metastatic cases among N/M+ patients also showed a similar (although non-significant) trend across clinical settings: 6.3% (3/48) in first-time BCR; 12.7% (9/71) in BCR after SRT; and 21.9% (7/32) in patients with BCP. 

### Comparison of Analog versus Digital PET/CT

**Figure 1:**
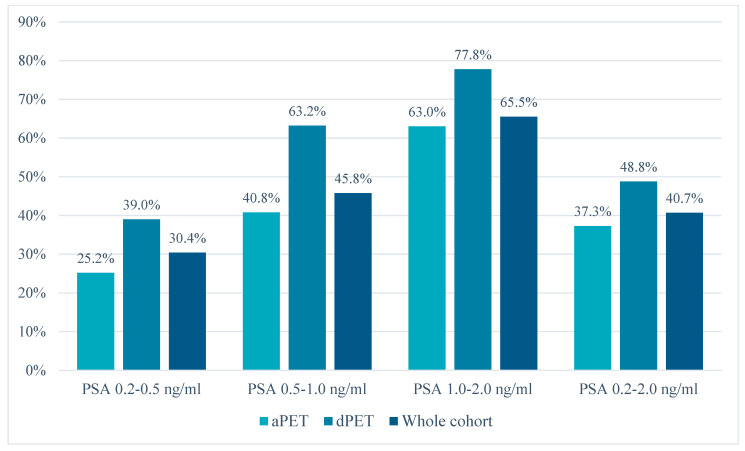
PET/CT detection rates stratified by PSA value and scanner type (analog vs. digital).

Regarding the PET/CT scanner technology, 311 patients underwent ^68^Ga-PSMA-11 imaging using an analog scanner (aPET) while 129 on a digital system (dPET). As shown in Table 1, the cohorts were comparable in terms of baseline clinical parameters (T-stage ≥ 3a, ISUPgrade ≥ 3, radical treatment, time-to-recurrence, PSA doubling-time/velocity, adjuvant/salvage treatments, clinical settings), except for a higher number of pN1 cases in the dPET cohort and a higher rate of positive resection margins (pR1) in the aPET cohort. Finally, a slightly lower median PSA value at PET scan was found for the dPET cohort (0.33 [IQR: 0.26–0.61] vs. 0.55 [IQR: 0.40–0.85] ng/mL, *p* < 0.01). Despite the lower median PSA value, dPET was associated with a significantly higher number of positive scans compared to aPET in the whole cohort (48.8% [63/129] vs. 37.3% [116/311], *p* = 0.03). The detection rate of the two scanner types was compared across different PSA value ranges, resulting significantly higher in both PSA ranges 0.2–0.5 ng/mL (39.0% [32/82] vs. 25.2% [34/135], *p* = 0.03) and 0.5–1.0 ng/mL (63.2% [24/38] vs. 40.8% [53/130], *p* = 0.02), but not for PSA ≥ 1.0 ng/mL. Data regarding the detection rate stratified by PSA value range and PET scanner type are presented in Figure 1 and Table 2.

**Figure 2:**
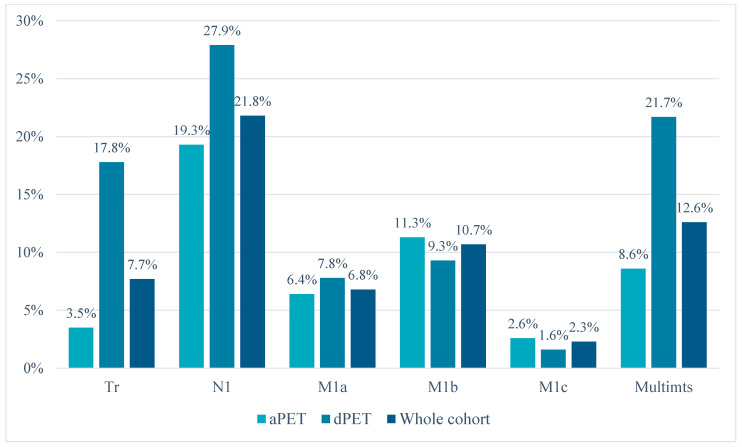
Distribution of PSMA-PET/CT pathologic findings (according to the miTNM definition) and proportion of multi-metastatic spread (among locoregional and/or distant metastatic cases) for analog and digital PET scanners.

Regarding lesion localization, dPET identified a higher number of cases with suspected local recurrence (miTr, 17.8% [23/129] vs. 3.5% [11/311], *p* < 0.01) or pelvic nodal metastases (miN1, 27.9% [36/129] vs. 19.3% [60/311], *p* = 0.05). The distribution of the detected lesions for each scanner type is reported in Figure 2 and Table 3. Furthermore, dPET also detected a higher number of multi-metastatic cases (>3 lesions) among N1/M1-positive scans (21.7% [10/46] vs. 8.6% [9/105], *p* = 0.03).

Overall, dPET identified a higher per patient median number of pathologic lesions (PSMA-RADS ≥ 3) (2 [IQR: 1–3] vs. 1 [IQR: 1–2], *p* = 0.001), while still presenting a significantly lower proportion of uncertain findings (PSMA-RADS 3) among all pathological lesions (24.4% [39/160] vs. 38.5% [60/156], *p* = 0.008), even when considering only miN1 lesions (10.8% [7/65] vs. 41.0% [25/61], *p* < 0.001). Moreover, despite the higher positivity rate of dPET, the number of patients with only uncertain findings was not significantly different between dPET and aPET (dPET 7% [9/129] vs. aPET 9% [28/311]).

**Figure 3:**
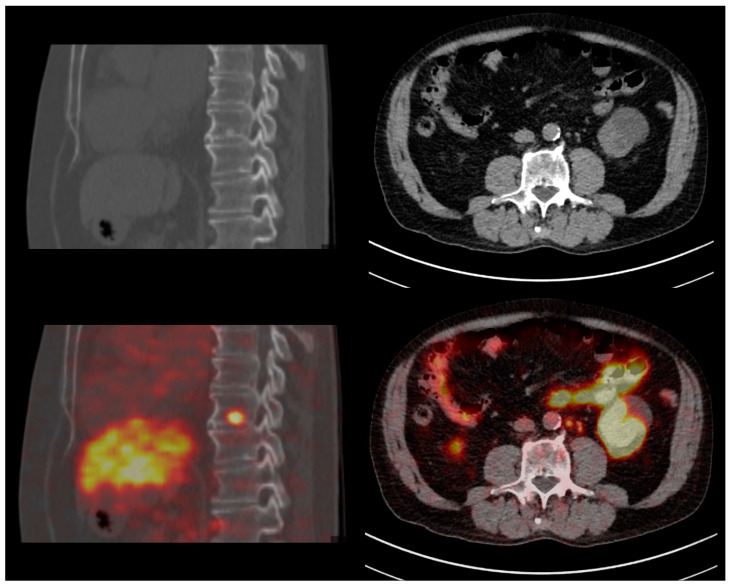
Clinical cases—Restaging of two early-recurrent prostate cancer patients with digital PSMA-PET/CT. Left column: 73 yo patient with Gleason 9 (4 + 5) pT3bN1 prostate cancer, treated with radical prostatectomy + linfadenectomy (03/2017) and salvage RT (07/2017); biochemical recurrence with PSA 0.23 ng/mL. PSMA-PET/CT identified a vertebral (D9) osteoblastic metastasis with intense tracer uptake. Right column: 75 yo patient with Gleason 9 (4 + 5) pT3bN1 prostate cancer, treated with radical prostatectomy and linfadenectomy (01/2019); biochemical recurrence with PSA 0.22 ng/mL. PSMA-PET/CT identified multiple extra-pelvic (para-aortic) subcentimeter nodal metastases (maximum diameter: 5 mm).

Regarding PET semi-quantitative analysis, the median SUVmax values of the lesions (PSMA-RADS ≥ 3) and reference regions (blood pool, liver, salivary glands) were higher at dPET compared to aPET: lesions SUVmax (5.0 [3.3–9.0] vs. 3.1 [2.0–5.7]), blood pool SUVmax (2.1 [1.8–2.3] vs. 1.3 [1.2–1.6]), liver SUVmax (7.6 [6.4–9.3] vs. 5.9 [5.0–6.9]), salivary glands SUVmax (19.8 [15.2–23.1] vs. 14.3 [11.9–18.5]) (all *p*-values < 0.001). Finally, the median lesion-to-blood-pool SUVmax ratio was comparable (even after PSMA-RADS stratification) between dPET and aPET (2.6 [1.7–4.3] vs. 2.3 [1.5–4.0], *p* = 0.2), but dPET identified nodal metastases with a smaller maximum diameter compared to aPET (PSMA-RADS 4 miN1/M1a: 5 [IQR: 4–7] vs. 7 [IQR: 5–8] mm, *p* = 0.002). Figure 3 illustrates the findings from two PCa patients restaged with PSMA dPET for early BCR (PSA 0.2 ng/mL).

## 4. Discussion

Our study investigated a cohort of hormone-sensitive prostate cancer (HSPC) patients at an early stage of disease recurrence after radical treatment (median PSA at PET scan 0.51 [IQR: 0.33–0.80] ng/mL), thus representing a potential low tumor burden population eligible for salvage treatment. Although PET/CT imaging with PSMA-targeted radiopharmaceuticals has shown remarkable diagnostic capabilities in PCa BCR patients, accurate lesion localization in the initial stages of disease recurrence still poses a significant diagnostic challenge. Novel SiPM-based digital PET/CT scanners have marked a significant advancement in nuclear medicine molecular imaging, showing superior sensitivity and better image quality compared to analog PET/CT systems [34] and holding promise for an earlier and more accurate restaging of oligometastatic patients eligible for SABR/SBRT treatments. Therefore, the aim of this study was to investigate whether the favorable characteristics of digital PET (dPET) compared to previous generation analog systems (aPET) could translate into improved disease localization in hormone-sensitive prostate cancer (PCa) patients with early biochemical recurrence/persistence (BCR/BCP) after radical treatment.

In our study, dPET showed a significantly higher detection rate compared to aPET, especially at lower PSA values (<1 ng/mL). dPET also identified a significantly higher number of pathologic findings (PSMA-RADS ≥ 3) per patient and more multi-metastatic cases (>3 lesions) compared to aPET. Previous literature studies have compared the performance of digital and analog PET/CT in prostate cancer patients [35,36,37,38,39]. Specifically, Alberts et al., in a retrospective evaluation of two matched cohorts of BCR Pca patients undergoing ^68^Ga-PSMA-11 imaging (aPET: 88, dPET: 88), found that dPET identified a greater number of pathological findings. In line with our results, dPET showed a significantly higher detection rate in the overall cohort (84% vs. 73%) as well as in the lower PSA ranges (<0.5 ng/mL and 0.5–2.0 ng/mL) [35]. Similar results were also obtained in a following retrospective study by the same authors, in which dPET showed a superior sensitivity and a higher true positive rate at follow-up compared to aPET [36]. A prospective study by Duan et al. on a lower sample size (*n* = 58) also reported dPET to be able to identify additional sites of recurrence, with higher sensitivity and specificity at lower PSA ranges, despite not reaching statistical significance [37]. The comparative performance of dPET and aPET in Pca patients has also been evaluated using other radiotracers such as ^18^F-DCFPyL, where dPET outperformed aPET in subjects with biochemical failure and PSA < 0.5 ng/mL (69% vs. 37%) while no significant differences were found at higher PSA values [38]. Contrary to PSMA-based radioligands, dPET was not able to achieve a superior diagnostic performance when using ^18^F-Fluorocholine for pelvic nodal staging of intermediate to high-risk PCa patients using histopathology as reference standard [39]. Indeed, the overall diagnostic accuracy of digital PET/CT molecular imaging is also influenced by the intrinsic performance of the administered radiotracer, and PSMA-based radioligands have been shown to outperform other tracers such as choline and fluciclovine [5,6]. Based on the above evidence, the improved technical performance of dPET could be best leveraged in the setting of early-BCR with low PSA values (<0.5 ng/mL or <1 ng/mL), where the higher sensitivity of dPET could potentially translate into an improved detectability of lesions with smaller dimensions and/or lower tracer uptake. On the contrary, in patients with higher PSA levels, the higher tumor burden and the higher degree of PSMA tracer uptake increase the chance that disease localizations are also detected by analog scanners despite their lower sensitivity, thus resulting in a higher number of positive scans at aPET and a detection rate comparable to dPET.

In our study, nodal metastases identified at dPET had a slightly smaller median diameter compared to aPET. Accordingly, previous literature studies on both humans and phantoms have reported on the capability of dPET to detect smaller lesions thanks to a consistently better signal-to noise ratio (SNR) [40,41], a higher sensitivity and a more stable TOF resolution and TOF-related gain even in the upper range of count rates observed in clinical routine [34,42]. Besides hardware improvements, other factors that could potentially contribute to enhanced dPET lesion detectability include novel reconstruction algorithms such as the Bayesian penalized likelihood reconstruction (BPL) which has been shown to improve the quantification and detectability of sub-centimetric spheres compared with OSEM-based reconstruction [43]. Despite this evidence, a previous study did not show a significant difference in lesion size between dPET and aPET, although reporting a higher tumor-to-background ratio (TBR) for dPET, therefore resulting in an improved lesion detectability nonetheless [35]. This aspect should also be further investigated using other PSMA tracers, such as ^18^F-DCFPyL [38].

Since our study was not based on an intra-patient comparison but on matching cohorts, a limited comparison of SUVmax values between dPET and aPET could be performed. dPET was associated with higher lesion SUVmax values, in line with previous intra-patient studies with other tracers which also documented higher SUVmax values for dPET compared to aPET [44,45]. The median SUVmax values of the reference regions (blood pool, liver, salivary glands) at dPET were also higher in our study, thereby resulting in comparable lesion-to-blood-pool SUVmax ratios between aPET and dPET. However, the identification of additional lesions with smaller diameters and lower absolute SUVmax values at dPET could have lowered the median lesion-to-blood-pool SUVmax ratio of the dPET group, thus resulting in the absence of statistical significance; this limitation could be overcome with an intra-patient study design.

Despite the higher overall detection rate of dPET, the prevalence of uncertain findings (PSMA-RADS 3) in our study was found to be significantly lower for dPET than aPET. A previous study by Alberts et al. on a smaller cohort has also shown a lower rate of uncertain lesions at dPET with ^68^Ga-PSMA-11, without reaching statistical significance [36]. However, these results cannot be generalized to all PSMA radiotracers. In the case of tracers such as ^18^F-PSMA-1007, the higher sensitivity of dPET could potentially worsen the risk of pitfalls related to benign tracer uptake (e.g., unspecific bone findings). Indeed, a retrospective study by Grunig et al. in patients undergoing ^18^F-PSMA-1007 PET/CT reported unspecific bone findings to be significantly more frequent in dPET than aPET, with relevant implications for therapeutic management in 45% of cases [46].

Other potential benefits of dPET imaging include the possibility to reduce the scan time and/or the administered dose. Indeed, in our study the ^68^Ga-PSMA-11 injected activity was comparable between aPET and dPET, but the acquisition time was 40% lower for dPET (1.5 vs. 2.5 min/bed position). Despite the lower acquisition time, dPET achieved a higher overall detection rate. Similar efforts have been previously described in the context of prostate cancer imaging by four studies [47,48,49,50]. Specifically, Vierasu et al. investigated BCR PCa patients (*n* = 54, median PSA 1.66 ng/mL) with ^18^F-JK-PSMA-7 dPET leveraging a reduced dose and acquisition time (20% and 50%, respectively) and demonstrating a comparable diagnostic performance [47]. Similarly, Weber et al. also found that at a 3.5-fold reduced scan time for ^68^Ga-PSMA-11 dPET the per region detection rate was still 98% and the mean quantitative SUVmax deviation was ≤10% with only a 5% downstaging rate [48]. Other factors that could contribute to reducing the scan time or the injected dose include novel reconstruction techniques, such as the aforementioned BPL algorithms. Indeed, a retrospective study by Yang et al. demonstrated that total variation regularized expectation maximization (TVREM) improved the image quality of ^68^Ga-PSMA-11 dPET imaging compared to OSEM thus allowing for reduced scan times [49]. These results are in line with another retrospective study by Lindstrom et al. in biochemically-recurrent PCa patients which showed that regularized reconstruction by block-sequential regularized expectation maximization (BSREM) led to lower background variability (BV) compared to OSEM, improved target-to-background ratio and signal-to-noise ratio, and a 50–75% decrease in scan time while keeping the BV below the 15% threshold [50].

Finally, digital PET/CT imaging also holds potential for improved cost-effectiveness from both a patient and health-care perspective: indeed, the higher image quality and the more accurate lesion localization could allow for more effective personalized imaging-guided treatments (that reduce the costs and complications associated with ineffective treatments), as well as lower scan times and administered radioactive doses thus improving patient radiation protection [51].

### Limitations

Our retrospective study is not exempt from limitations. Indeed, a study design based on an intra-patient comparison would have been preferable since it would have allowed a direct comparison of the pathologic findings of each patient. However, the two cohorts of biochemically recurrent PCa patients had comparable clinical characteristics (Table 1). A similar approach based on a matched-pair comparison has also been previously reported in literature [35]. Although a selection bias could not be completely ruled out, despite a slightly lower median PSA value in the dPET cohort (0.33 [IQR: 0.26–0.61] vs. 0.55 [IQR: 0.40–0.85] ng/mL), dPET was still able to exhibit a superior diagnostic performance compared to aPET. Another potential limitation of this study is related to the scanning acquisition parameters and reconstruction algorithms. Indeed, these settings differed between the two scanners and could have influenced the results [52]. However, the retrospective design of our study reflects routine clinical experience, in which acquisition parameters and reconstruction algorithms are tailored to each scanner’s performance and manufacturer’s recommendations. Histological validation of PET/CT pathologic findings was not routinely feasible due to ethical and practical reasons; however, high positive predictive values for PSMA-PET have been already established by previous studies [8]. Finally, the data on dPET performance presented in this study were obtained using the ^68^Ga-PSMA-11 radiotracer, but further investigations with other PSMA-based radiopharmaceuticals should be performed before generalizing these results to other radiolabeled PSMA inhibitors. Despite these limitations, the enhanced performance of dPET holds promise for improving molecular imaging diagnostic accuracy and opens new perspectives for further investigations on novel technological developments such as intraoperative PET/CT imaging [53,54] and long axial field-of-view (LAFOV) PET/CT scanners [55]. Proper economic analyses will also be needed to evaluate the cost-effectiveness of dPET imaging in this setting: they will have to consider the costs and the utilities resulting from the impact of dPET higher restaging accuracy on patients’ management and prognosis.

## 5. Conclusions

^68^Ga-PSMA-11 digital PET/CT showed a superior performance compared to analog PET/CT in restaging prostate cancer patients with early biochemical recurrence/persistence, leading to a higher detection rate especially at lower PSA values (≤1 ng/mL). Moreover, digital PET/CT detected a higher number of pathological findings and allowed to identify nodal metastases with a smaller diameter, while also showing a lower rate of uncertain findings compared to analog PET/CT.

## Figures and Tables

**Table 1:** Characteristics of the study population (440 hormone-sensitive prostate cancer patients).

Clinical Features		Analog PET (*n* = 311)	Digital PET (*n* = 129)	
		Median [IQR]	Median [IQR]	*p*-Value
Age (years)		76 [70–80]	75 [68–79]	0.1
iPSA (ng/mL)		8.04 [5.96–13.00]	6.70 [5.20–10.00]	0.4
PSA at PET scan (ng/mL)		0.55 [0.40–0.85]	0.33 [0.26–0.61]	<0.001
PSAdt at PET scan (months)		8.30 [4.40–14.80]	7.40 [3.90–11.65]	0.1
PSAvel at PET scan (ng/mL/year)		0.50 [0.20–0.90]	0.30 [0.20–0.70]	0.09
**Clinical Features**		**Frequency % (*n*)**	**Frequency % (*n*)**	***p*-Value**
ISUP Grade (1–2 vs. 3–5)	1	13.0% (39/300)	7.3% (9/123)	0.2
	2	22.3% (67/300)	17.9% (22/123)	
	3	32.0% (96/300)	35.8% (44/123)	
	4	18.7% (56/300)	26.8% (33/123)	
	5	14.0% (42/300)	12.2% (15/123)	
pT stage	<3a	48.0% (145/302)	40.7% (50/123)	0.2
	≥3a	52.0% (157/302)	59.3% (73/123)	
pN stage	N1	9.4% (24/254)	20.6% (22/107)	0.005
R (margin)	R1	50.8% (122/240)	35.5% (33/93)	0.01
Time to PSA relapse (months)	<12	25.3% (78/308)	34.1% (44/129)	0.8
Primary therapy	RP ± LND ± adjuvant RT	97.1% (302/311)	95.3% (123/129)	0.4
	Primary RT	2.9% (9/311)	4.7% (6/129)	
Adjuvant RT	RT received	15.4% (48/311)	9.4% (12/128)	0.1
Adjuvant ADT	ADT received	11.9% (37/311)	7.0% (9/128)	0.2
Clinical stage of PSA failure at PSMA PET/CT	First BCR	46.6% (145/311)	48.8% (63/129)	0.08
	PSA relapse after prostate-bed SRT	40.8% (127/311)	31.8% (41/129)	
	BCP after RP	12.5% (39/311)	17.4% (25/129)	

**Table 2:** PET/CT detection rates stratified by PSA value and scanner type (analog vs. digital).

PET Scanner Type	PSA Range 0.2–0.5 ng/mL	PSA Range 0.5–1.0 ng/mL	PSA Range 1.0–2.0 ng/mL	PSA Range 0.2–2.0 ng/mL
aPET	25.2% (34/135)	40.8% (53/130)	63.0% (29/46)	37.3% (116/311)
dPET	39.0% (32/82)	63.2% (24/38)	77.8% (7/9)	48.8% (63/129)
aPET + dPET	30.4% (66/217)	45.8% (77/168)	65.5% (36/55)	40.7% (179/440)
*p*-value	0.03	0.02	0.47	0.03

**Table 3:** Distribution of PSMA-PET/CT pathologic findings (according to the miTNM definition) and proportion of multi-metastatic spread (among locoregional and/or distant metastatic cases) for analog and digital PET/CT scanners.

Scanner	Tr	N1	M1a	M1b	M1c	Multimts
aPET	3.5% (11/311)	19.3% (60/311)	6.4% (20/311)	11.3% (35/311)	2.6% (8/311)	8.6% (9/105)
dPET	17.8% (23/129)	27.9% (36/129)	7.8% (10/129)	9.3% (12/129)	1.6% (2/129)	21.7% (10/46)
aPET + dPET	7.7% (34/440)	21.8% (96/440)	6.8% (30/440)	10.7% (47/440)	2.3% (10/440)	12.6% (19/151)
*p*-value	< 0.001	0.05	0.7	0.6	0.7	0.03

## Data Availability

The data are not publicly available due to ethical and privacy reasons.
